# Commentary on heart valve inflow orifice diameter

**DOI:** 10.1093/icvts/ivad097

**Published:** 2023-06-14

**Authors:** Tirone David

**Affiliations:** Division of Cardiovascular Surgery, Peter Munk Cardiac Centre, Toronto General Hospital and University Health Network, Toronto, ON, Canada

**Keywords:** Heart valve surgery, Prosthetic heart valves, Bioprosthetic heart valves

In this issue of this journal, van Boxtel and colleagues [1] reported the results of a study whereby they geometrically measured the inflow orifice diameter (IOD) of 8 commercially available supra-annular bioprosthetic valves and concluded that ‘Valve packages should be labelled accurately and clearly for surgeons to make a well-informed choice. Currently essential information is missing, since intended position in relation to the annulus is not consistently marked on the packing boxes, and valve sizes are labelled incorrectly’. They are not the first one to show that valve sizes are not correctly labelled for the annular size but are the first ones to report on the geometric IOD of various bioprosthetic heart valves. Do surgeons care about the IOD of bioprosthetic heart valves or are more interested in the effective orifice areas of valves to be implanted?

Labelling of heart valve substitutes has been confusing since the introduction of the first man-made artificial heart valve, the Starr–Edwards ball valve. That is the reason manufacturers provide them with sizers to measure the annulus where they are to be implanted. This labelling problem is not only with supra-annular bioprosthetic heart valves used for aortic valve replacement but also with mechanical heart valves that are implanted in all positions in the heart as well as with annuloplasty devices used for heart valve repair. This issue is so important that the 3 major European and American cardio-thoracic surgical societies endorsed a document prepared by a panel of experts on heart valve substitute sizing [2].

The ideal prosthetic heart valve substitute should offer minimal impedance to blood flow and this can often be attained by knowing the estimated effective orifice area of each valve type and size and matching to the patient’s body surface area. The initial problem is not the IOD but rather the mislabelling by the various manufacturers and the great variability in sizes for any given size and the effective orifice areas [3, 4]. For one, it is unwise to measure the size of the annulus with a sizer of a valve manufacturer and implant a valve from a different manufacturer. In addition, one is expected to know the estimated effective orifice areas of valves used and try to implant a valve that reduces the risk of patient-prosthesis mismatch in both the aortic and mitral positions. Finally, since the advent of transcatheter valve implantation, the IOD is also important because small bioprosthetic heart valves may be unsuitable for a valve in valve when degenerative changes occur and reintervention in a failing bioprosthetic valve is being considered.

The suggestions proposed by van Boxtel and Mariani [1] should be required from heart valve manufacturers, but surgeons have to remain knowledgeable of all limitations of heart valve substitutes and aware of marketing strategies used by their makers.
